# GP problems when communicating with patients about their alcohol drinking: an investigation in Florence (2010-2012)

**DOI:** 10.1186/1940-0640-8-S1-A3

**Published:** 2013-09-04

**Authors:** Allaman Allamani, A Centurioni, S Innocenti, A Mustur, G Findazini, M Puliti

**Affiliations:** 1 Italian Agency for Health Services No. 2, "Isontina", Tuscan Region, Gorizia, Italy

## 

The perception of Italian GPs on alcohol problems has changed in recent decades, but it is still inclined to make a too clear-cut distinction between normal drinkers without any problem and hopeless alcoholics, according to an ongoing cultural tradition. On the other hand, according to consistent observation during the last 25 years, the identification of risky drinkers among clients has been as low as 4-11%, and of alcoholics between 0.8 and 2%. Understanding the obstacles that GPs encounter when they communicate with patients about the issue of alcohol can provide appropriate information tools to improve GP's ability to identify risky drinkers and to motivate those identified to reduce their drinking behaviour. The BIQ questionnaire, previously used by Struzzo in Italy, was administered to 158 GPs in the area of Florence who attended a one-day alcohol training course, prior to the start of the training. Six such courses were carried out during the period 2010-2012. 45.7% of participants recognized as a limit for at risk drinking for males of 30-40 g of alcohol per day, and 20.4% identified 20-30 g of alcohol per day for females (70.6% of whom set this limit at 10-20 g per day, suggesting a more restrictive approach to female patients regardless the gender of the physician). 47.7% said they have no problem addressing the issue of alcohol with their patients, while 52.7% said they had little or no effect on changing their patients’ drinking behaviour. A need for training in early identification and brief intervention was felt by most GPs (over 90%), with a minority (14%) indicating that an economic incentive is necessary to implement brief intervention. As to GP lifestyles, 81% of physicians reported drinking at least one alcoholic beverage during the last year, and 25% smoked (above the mean for Tuscany and Italy).

